# Articular cartilage delamination at eight years following cellular-based repair procedures: a case reports

**DOI:** 10.1186/s40634-022-00527-2

**Published:** 2022-09-07

**Authors:** Alberto Gobbi, John G. Lane, Macarena Morales, Riccardo D’Ambrosi

**Affiliations:** 1 O.A.S.I. Bioresearch Foundation Gobbi Onlus, Milan, Italy; 2 Department of Orthopaedic Surgery, University of California, San Diego, San Diego, CA USA; 3 IRCCS Istituto Ortopedico Galeazzi, Milan, Italy; 4 Dipartimento Di Scienze Biomediche Per La Salute, Università Degli Studi Di Milano, Milan, Italy

## Abstract

This report describes two cases of late cartilage delamination in two young adults after two different autologous cell-based techniques for cartilage restoration: 1. Matrix-assisted autologous chondrocyte implantation (MACI) and 2. Hyaluronic acid-bone marrow aspirate concentrate (HA-BMAC). Both cases demonstrate that even in patients who do not present with any ongoing symptoms after primary surgery, a cellular-based graft’s subsequent delamination can occur later. It is possible that regardless of the technique used or the time passed since the surgery, a graft failure may occur at some level, causing delamination of a previously asymptomatic cartilage restoration graft and a traumatic event with long-term follow-up. Surgeons must be alert to this injury and describe histologic findings to determine where failure occurs.

## Introduction

Cartilage injuries are frequent, with 60 to 66% of patients being noted to have articular defects when undergoing arthroscopy [3]. These defects produce limitations in daily activities, work, recreational activities, and sports. Full-thickness chondral damage has an insufficient self-regenerative capacity; without suitable treatment, these defects can progress to degenerative joint disease [4, 6, 22]. Numerous techniques for repair are currently available, including two-stage autologous chondrocyte implantation (ACI, MACI), bone marrow stimulation (BMS), osteochondral autograft transplantation (OAT) and osteochondral allograft transplantation (OCA) [10, 15, 16]. Bone marrow aspirate concentrate combined with a biologic scaffold of hyaluronic acid (HA-BMAC) has demonstrated good to excellent clinical outcomes at long-term follow-up with the ability to fill defects with well-integrated repair tissue [8–12]. All these techniques have their respective indications and potential complications. Cellular techniques such as ACI, MACI and HA-BMAC repair the articular surface by incorporating the cellular matrix onto the subchondral plate [19], which often leads to restoration of the joint surface. However, graft delamination is a possible cause of failure. This has been noted in prior studies with the separation of the neocartilage tissue from the underlying subchondral bone due to shearing forces that cause these two layers to separate [20]. Different pathological conditions occur with cellular-based cartilage repair, including delamination, detachment, and graft hypertrophy. We would like to focus on the incidence of delamination of cartilage grafts in this publication. Delamination is the separation of articular cartilage from the subchondral bone at the tidemark level, parallel to the joint surface [23]. This is present with an intact knee where the articular cartilage surface remains intact initially. However, the natural history results in articular cartilage breakdown with resultant chondral flaps, full-thickness defects, and loose bodies. Conceptually, this same pathologic process can happen in cartilage restoration, where shearing of the regenerative tissue from the subchondral bone and a failure in the incorporation with the surrounding cartilage can occur. Delamination of the graft most frequently occurs during the first 12 months [16].

## Case presentation

### Case 1

**Fig. 1:**
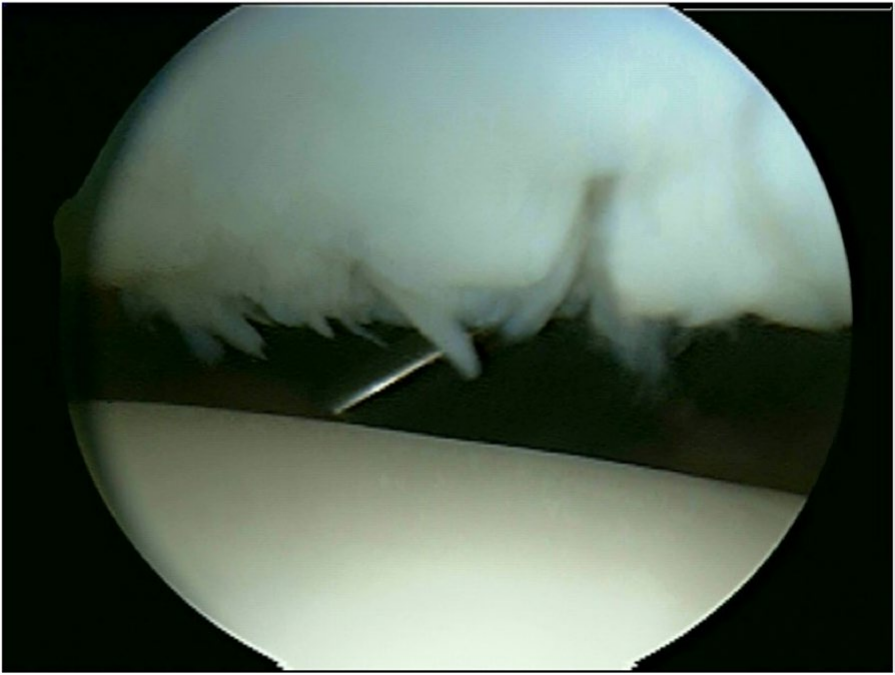
Patellar cartilage lesion in the entire lateral facet, grade 4 ICRS. 113 × 85 mm.^2^ (150 × 150 DPI)

**Fig. 2:**
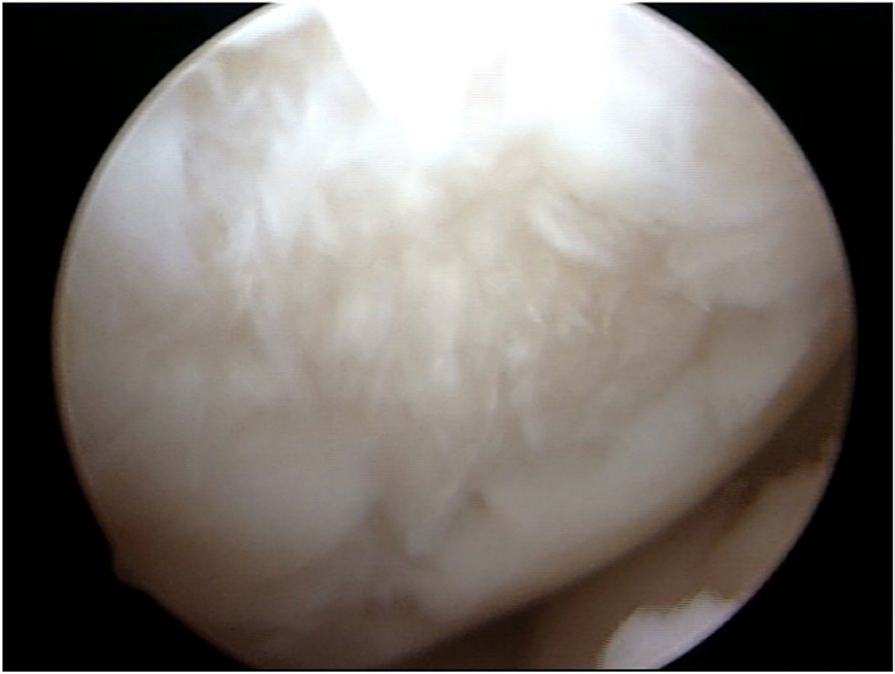
Medial femoral condyle cartilage lesion, 3.0 × 2.5 cm, with a well-contained margin. 113 × 85 mm.^2^ (150 × 150 DPI)

A 34-year-old man (height 175 cm; weight 76 kg; Body Mass Index [BMI] 24.8), involved in a sports activity (University of California at Los Angeles (UCLA) Activity level rating scale 6 [2]), had significant knee pain with patellofemoral crepitus and pain along the medial tibiofemoral joint line. No surgery had ever been performed on the affected knee. After a thorough preoperative evaluation, the patient was diagnosed with a chondral defect of the medial femoral condyle and the patella, with a normal lateral compartment and knee alignment. Arthroscopy was performed and revealed an isolated degenerative lesion on the lateral facet of the patella (International Cartilage Repair Society (ICRS) Classification Grade 4 [1]) (Fig. 1) with an intact and well-contained medial femoral condyle lesion (ICRS Grade 3-B) (Fig. 2). The lateral compartment was healthy. A cartilage biopsy was obtained for subsequent autologous chondrocyte implantation. Samples were harvested from the non-weight-bearing area of the intercondylar notch at the junction of the main weight-bearing zone of the medial and lateral condyles of the femur and the articular surface of the patella. The biopsy site was located approximately 2 mm from the cartilage border after the cartilage integrity at the biopsy site had been assessed [18].

We performed an autologous chondrocyte procedure seven weeks later via a medial peripatellar arthrotomy. The medial femoral condyle defect was a contained lesion approximately 30 mm by 25 mm. The patellar lesion was approximately 35 mm × 16 mm. The defects were prepared for cartilage repair, where a scalpel and ring curette were used to remove the diseased cartilage from the healthy subchondral bone. A Bio-Gide (resorbable bilayer porcine collagen membrane, Geistlich) was then cut in the usual fashion and sutured in place with the application of fibrin glue. Autologous chondrocytes were implanted under the Bio-Gide in the patella and medial femoral condyle lesions. After 13 months, the patient developed postoperative arthrofibrosis, and a new arthroscopy was performed 15 months after the first surgery for lysis of adhesions. Early onset OA may be a risk factor or indicator for developing arthrofibrosis after injury or surgery [21].

**Fig. 3:**
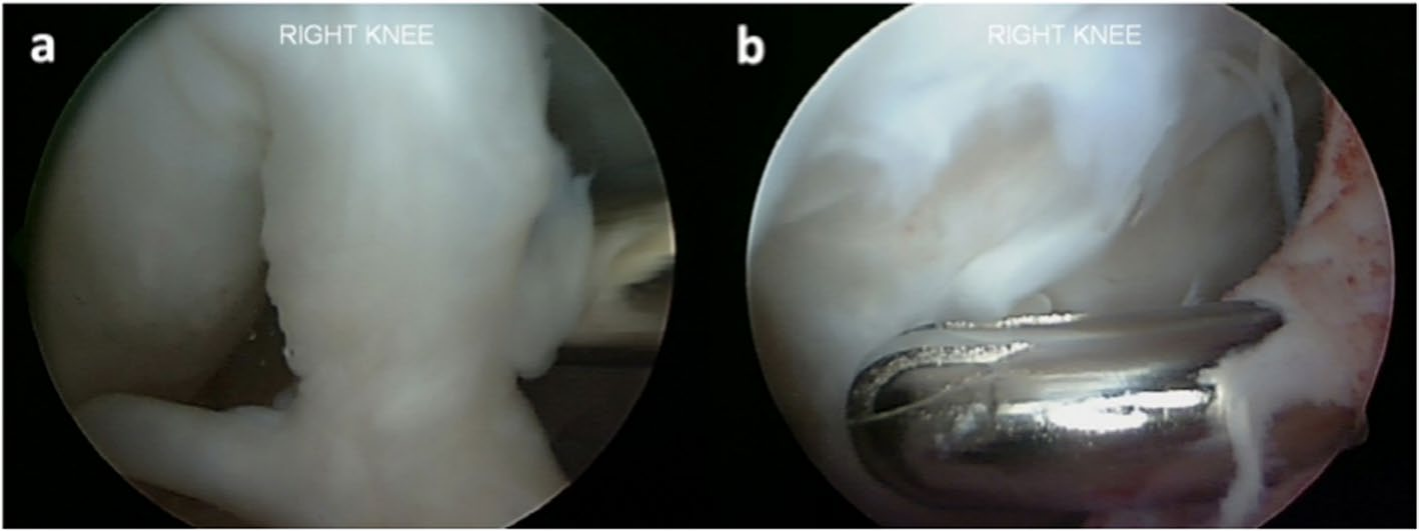
**a** Fibrous band tissue over the cartilage repair zone in the medial femoral condyle, 4 mm in thickness. **b** Chondroplasty was performed, removing the scar tissue from the joint portion using a shaver. 451 × 168 mm.^2^ (144 × 144 DPI)

**Fig. 4:**
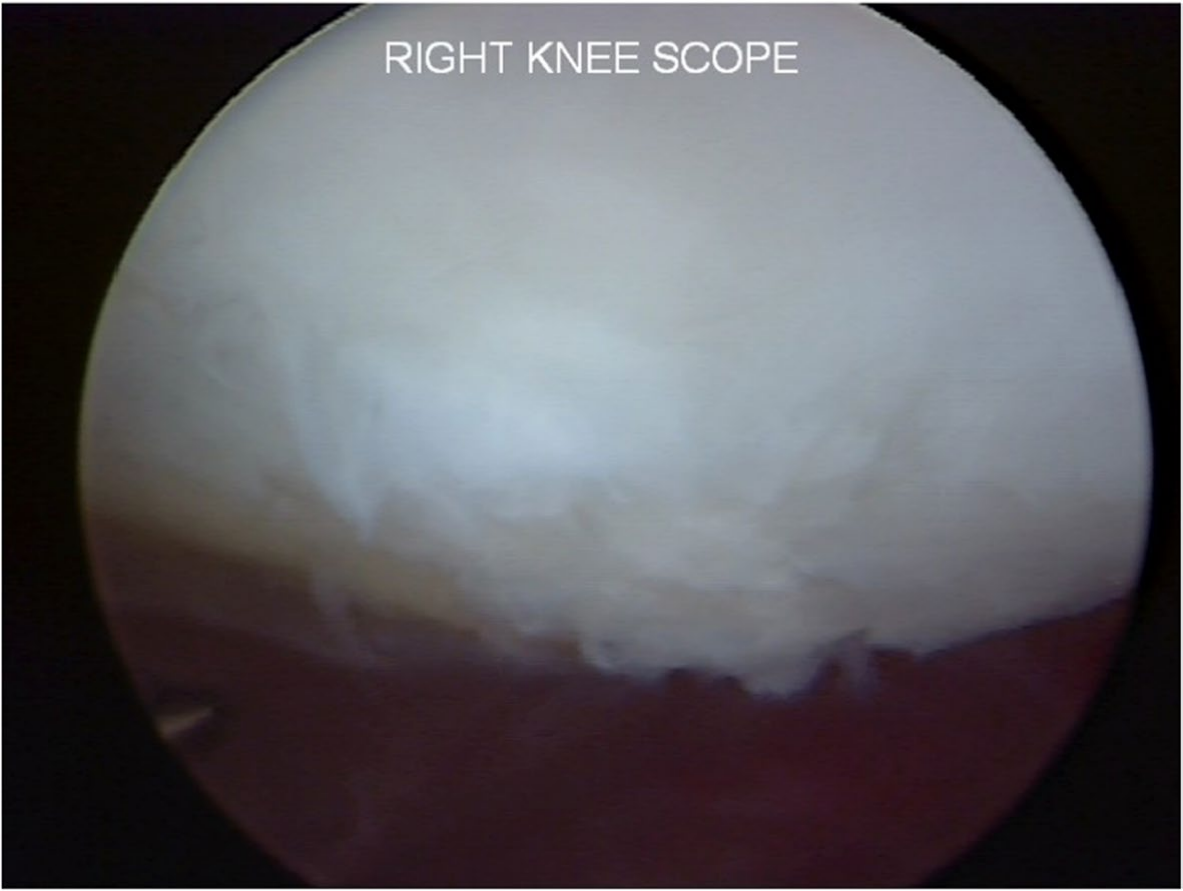
Grade 2 chondral injury in the lateral facet of the patella

During the procedure, firm cartilage-like tissue was identified in the patellofemoral and medial tibiofemoral joints. Debridement at the site of the graft area was performed as it was intact with graft hypertrophy but with no evidence of integration failure. The medial compartment did have a fibrous tissue layer over the cartilage repair site (Fig. 3a). A superficial chondroplasty was performed (Fig. 3b). After 8 years of being asymptomatic (UCLA 6), since the first surgery, the patient was involved in an altercation, falling directly onto the same injured knee with the acute onset of symptoms. The patient had trouble weight-bearing after this injury, used crutches and underwent diagnostic knee arthroscopy. The lateral facet of the patella showed a grade 2 lesion according to ICRS (Fig. 4).

**Fig. 5:**
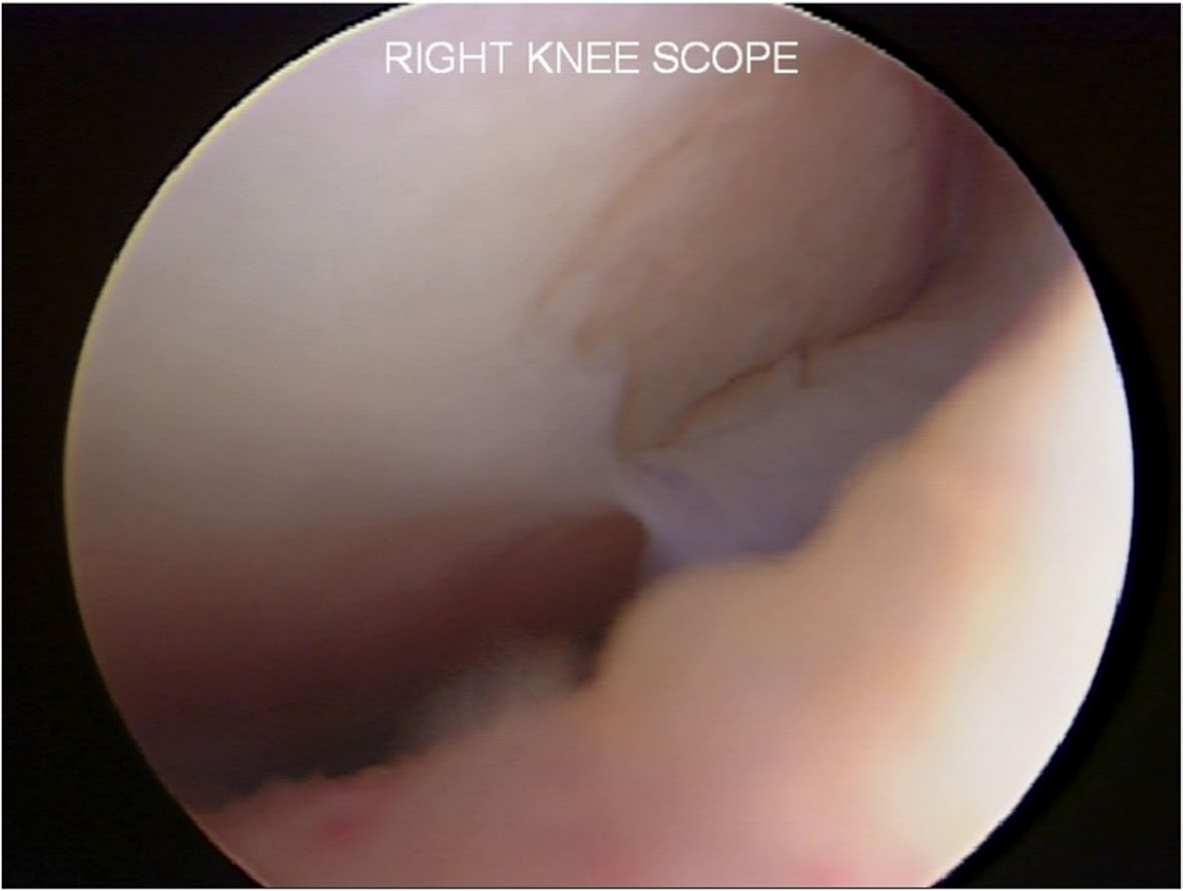
Complete delamination of a large chondral defect approximately 4 × 3 cm.^2^

**Fig. 6:**
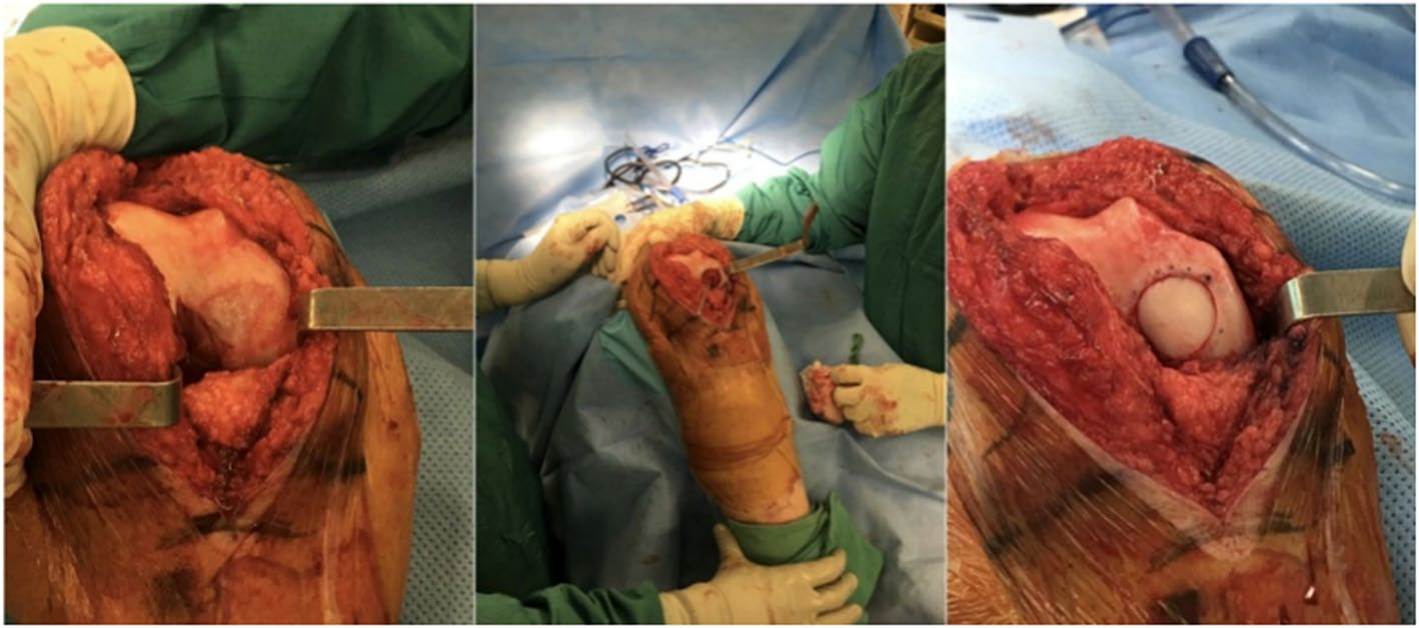
After acute delamination was demonstrated, osteochondral allograft was applied to the defect in the medial femoral condyle. 439 × 193 mm.^2^ (144 × 144 DPI)

**Fig. 7:**
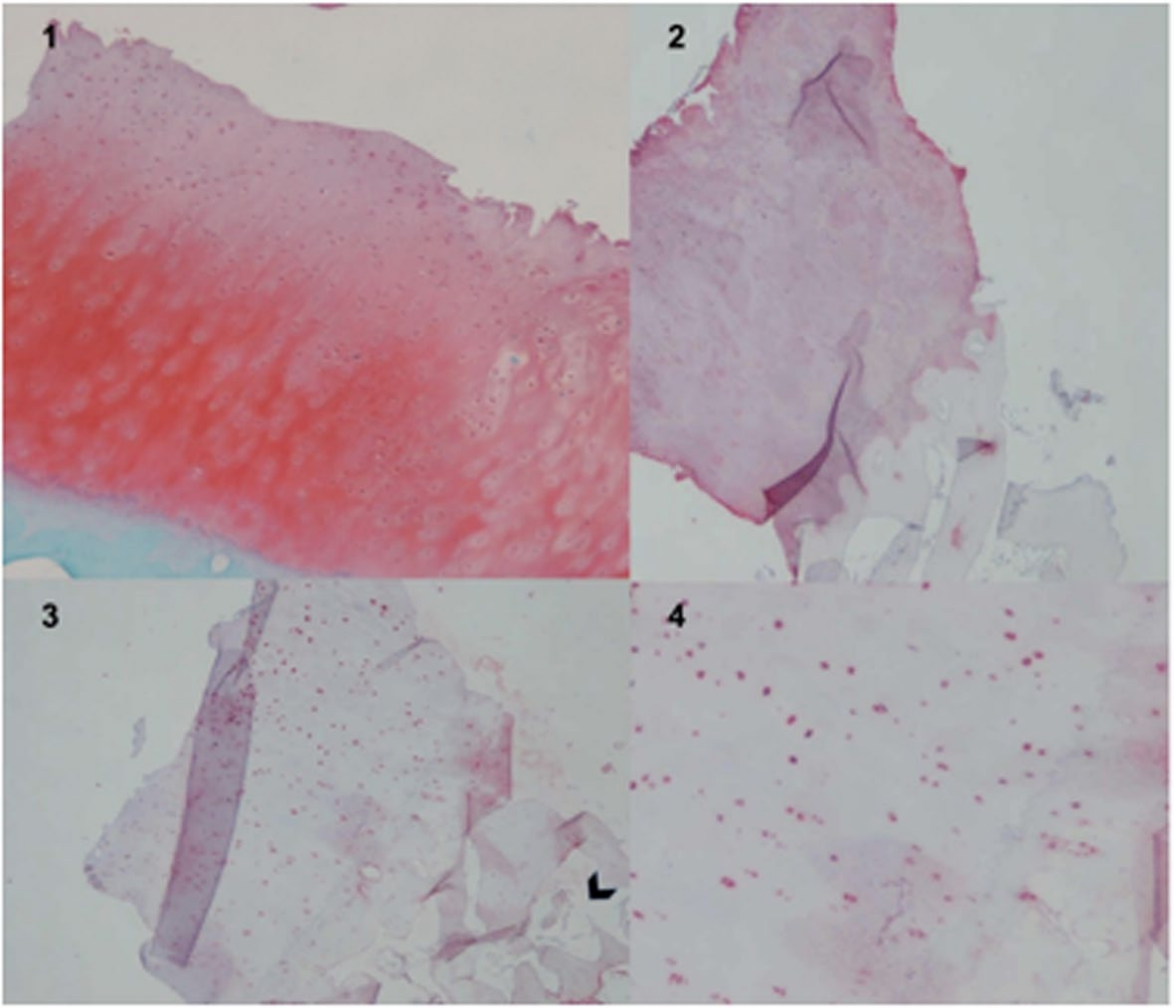
Safranin-O/Fast Green highlights the presence of a fairly organised cartilage structure (**1**). Immunohistochemical evaluation for type II collagen showed positivity, especially at the level of the extracellular matrix (**2**). Immunohistochemical evaluation for type I collagen showed positiveness at the cellular level. Delamination is present above the tidemark, black arrow. (**3**,**4**). 370 × 317 mm.^2^ (144 × 144 DPI)

On the medial femoral condyle, in the same location as the previous lesion, there was complete delamination of a significant chondral defect, approximately 4 cm × 3 cm (Fig. 5). The chondral flap was removed, and we subsequently performed an OCA on the defect. (Fig. 6). The histological exam of the delaminated tissue showed a fairly organized cartilage structure, and despite the surface not being smooth, the cellular components were present and had a normal distribution (Fig. 7).

### Case 2

**Fig. 8:**
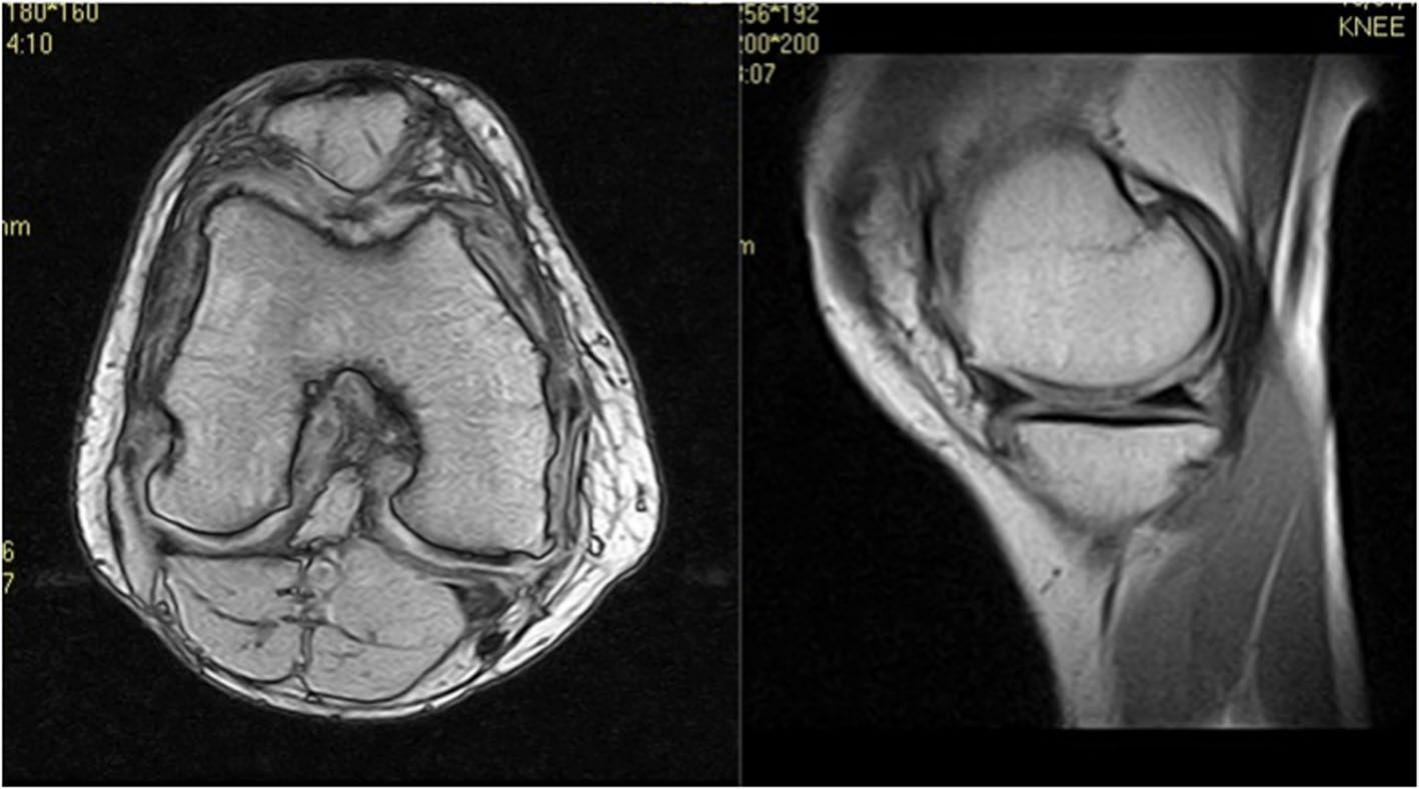
Patient MRI with a grade 4 cartilage lesion in the trochlea, patella and medial femoral condyle. 395 × 219 mm.^2^ (144 × 144 DPI)

A young man, 32 years old (height 181 cm; weight 85 kg; BMI 25.9), skis, swims and runs as a part of his sports activity (UCLA 6/7), noted pain in the anterior region of the knee and the medial compartment. No surgery had ever been performed on the affected knee. A grade 4 (according to ICRS) cartilage lesion diagnosis was noted in the central area of the right knee’s trochlea, patella, and medial femoral condyle (Fig. 8). The medial femoral condyle defect was a contained lesion approximately 32 mm × 27 mm. The patellar lesion was approximately 33 mm × 18 mm, while the trochlear lesion was about 28 mm × 20 mm.

**Fig. 9:**
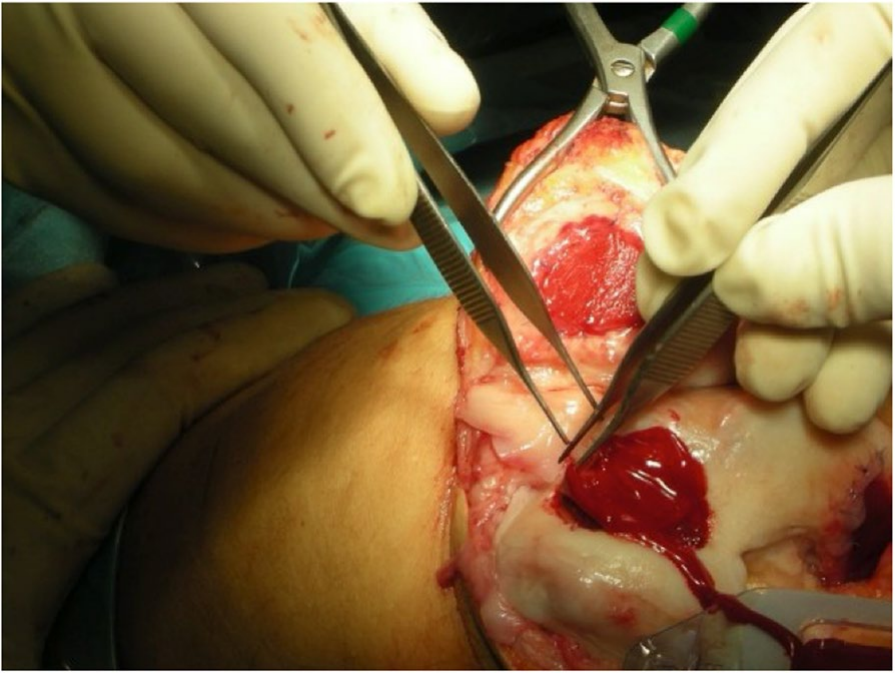
Cartilage repair with Hyaluronic Acid Scaffold plus BMAC (Bone Marrow Aspirate Concentrate) and fibrin glue in the trochlea, medial femoral condyle and patella

**Fig. 10:**
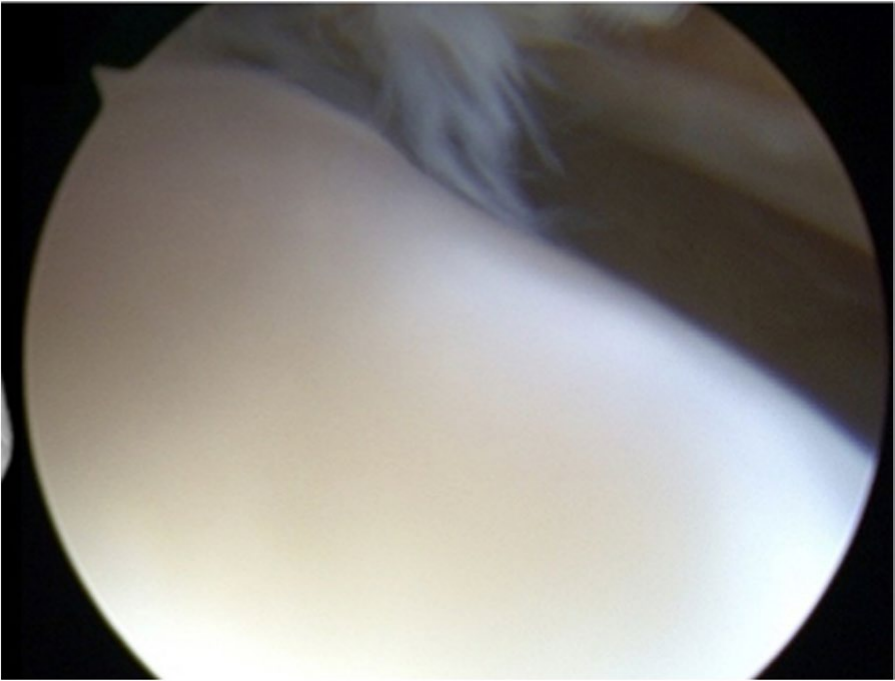
Second-look arthroscopy revealed a healed patellar lesion and fibrillations of the patella (Grade II according to ICRS classification). 324 × 149 mm.^2^ (144 × 144 DPI)

**Fig. 11:**
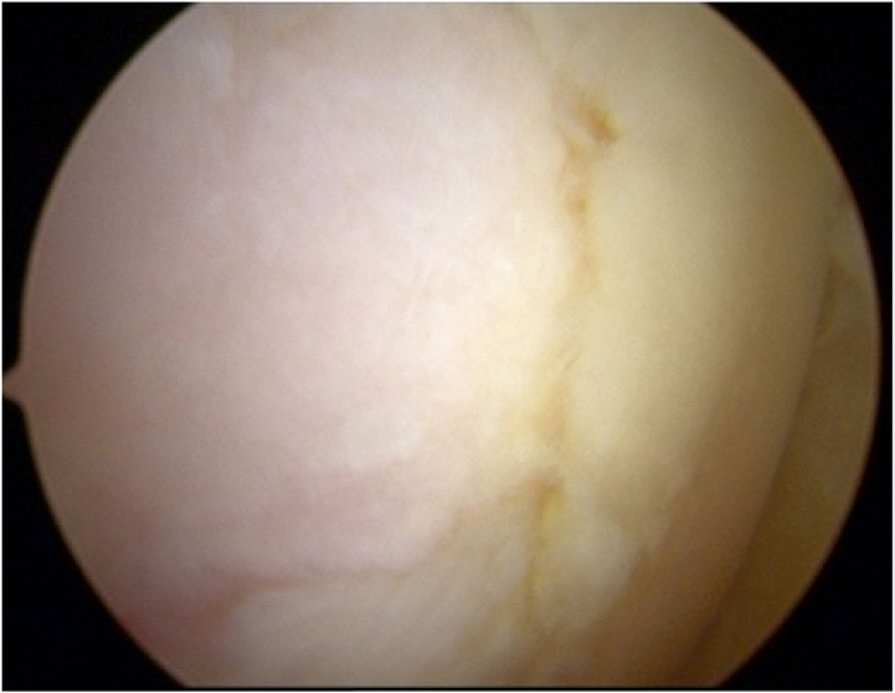
The arthroscopic view shows the delamination of the previous HA-BMAC scaffold with a size of 38 × 18 mm.^2^

**Fig. 12:**
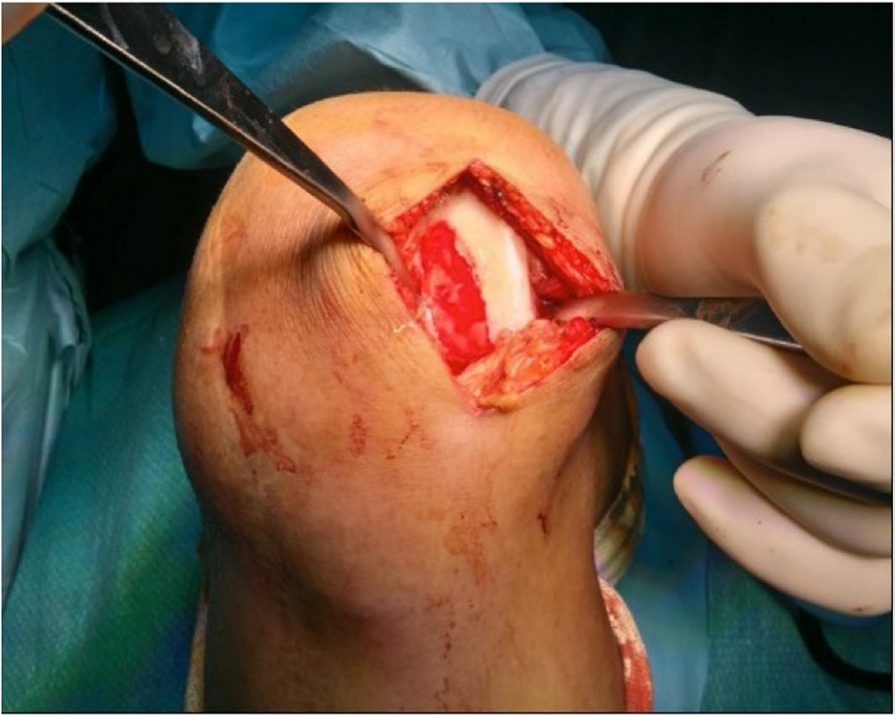
The chondral lesion was delaminated, and a new cartilage repair with HA-BMAC was performed successfully

**Fig. 13:**
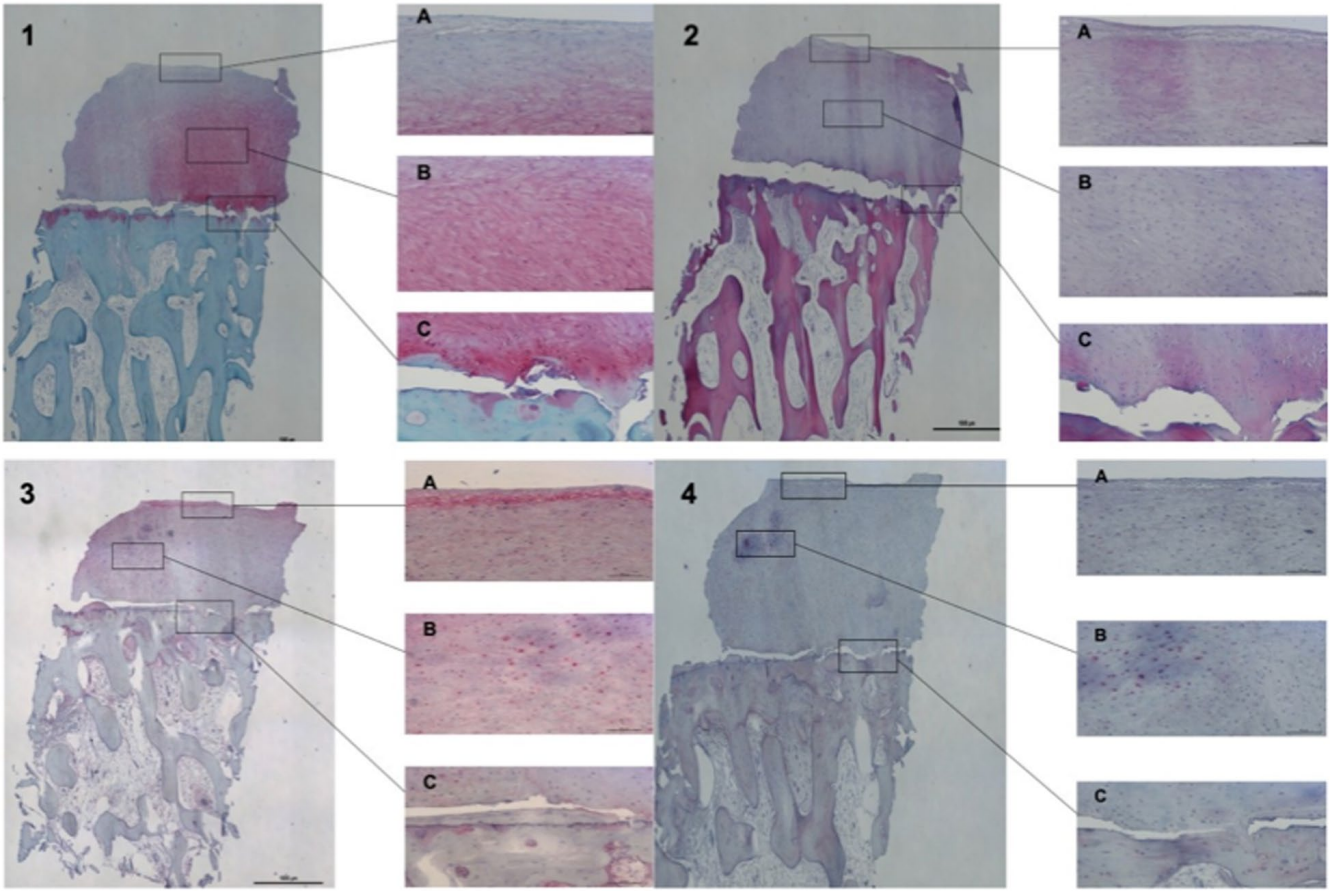
Safranin-O/Fast Green shows the presence of matrix proteoglycans in the middle and deep zones. Tidemark is also adjacent to subchondral bone (**1**). Haematoxylin–Eosin staining, where an organized cartilage tissue with a regular and smooth surface is seen (**2**). Immunohistochemistry with Collagen II is positive in the middle and deep zones, both in the extracellular and cellular (**3**). Collagen I is negative, except for some areas (**4**). All cuts demonstrate delamination the above tidemark. 475 × 317 mm.^2^ (144 × 144 DPI)

The patient underwent a one-stage cartilage repair with bone marrow aspirated from the iliac crest with a three-dimensional hyaluronic acid-based scaffold (Hyalofast, Anika Therapeutics) (HA-BMAC). A clot of bone marrow aspirate was placed into the full-thickness cartilage defect and prepared with stable vertical walls and intact subchondral bone. It was covered with a HA scaffold and sealed with fibrin glue (Fig. 9) [9, 11, 12]. Additionally, due to patella mal-tracking, an antero-medialization of the tibial tubercle was performed (tibial tuberosity to trochlear groove distance [TT-TG] 21 mm; J-Sign positive; Patello-femoral grind test positive) [5]. The patient performed well with no pain and symptoms and returned to sports activities a year after the operation. Eight years after surgery, he experienced a knee sprain while running. A new MRI demonstrated a chondral defect in the medial femoral condyle. Arthroscopy revealed excellent healing in the middle of the trochlea and an improved patella with fibrillations (Grade II according to ICRS classification) (Fig. 10). However, a full-thickness medial femoral condyle chondral lesion, on the site of previous repair, was consistent with the initial defect measuring 38 × 18 mm (Fig. 11). The chondral lesion was debrided, and a new cartilage repair with HA and BMAC was performed with the usual technique (Fig. 12). A sample of the delaminated fragment was evaluated by histology, showing a smooth and regular surface and the presence of matrix proteoglycans in the middle and deep zones. Delamination is noted with the tidemark adjacent to the bone. By immunohistochemistry, collagen I found to be negative with the exception of small areas with positive cells, and collagen II is positive at extracellular and cellular levels, especially in the superficial and middle zones (Fig. 13).

## Discussion

In the presented cases, two active adult patients were asymptomatic for over 8 years after a cellular-based repair procedure until developing acute knee symptoms after a traumatic event. It is generally acknowledged that patients over 30 years of age have, in general, inferior outcomes and higher failure rates and a need for revision surgery [7]. In both cases, the patients were asymptomatic, and postoperative studies demonstrated a well-healed cartilage repair. Eight years later, both had a traumatic event occur to the knee with the development of cartilage delamination in the initially repaired zone. Failure after ACI has been defined by Gomoll et al., who considered a failure by MRI and arthroscopy [13]. He noted that the graft has a structural compromise associated with pain and mechanical symptoms that require revision surgery. However, these two patients had no antecedent symptoms before a second traumatic incident occurred, and they developed pain and swelling. Minas et al. reported that 25% of patients who underwent a first-generation ACI procedure had a failure. Of the 53 out of 210 patients treated with periosteum patch-covered ACI, in 12 cases, delamination was the cause of the failure (23%) [16]. The failure rate is low (a mean of 5.8%, at a mean follow up of 22 months), highest in the first generation ACI, with hypertrophy and delamination as the most frequent complications [14]. According to the US Food and Drug Administration study, delamination is one of the four main complications in these patients, with up to 22% reporting adverse events in patients treated with ACI [24]. The other complications include graft hypertrophy, deficient or inadequate fusion, and insufficient regenerative cartilage [17]. It is also important to note that the vast majority of patients that underwent ACI were over 30 years of age [7]. In the studies mentioned before, delamination presented early after surgery [14, 16, 17, 24]. It is associated with shearing forces in the early stages when the cartilage layer has not integrated with the subchondral bone [20]. It has been postulated to occur due to non-compliance in weight-bearing initially and with significant or uncontained defects [20]. In our cases, delamination occurred many years later. Despite what was thought to be the successful integration of the articular cartilage graft, a traumatic event caused these regenerated cells to delaminate. This demonstrates that incomplete graft healing can lead to late delamination in a subset of asymptomatic patients after the first cartilage repair surgery, even when the MRI or arthroscopy shows the opposite. We must alert other surgeons faced with this kind of injury in the future to consider histologic evaluation of any failed tissue.
